# Supplementary material for: Conditional chemoconnectomics (cCCTomics) as a strategy for efficient and conditional targeting of chemical transmission
Source: eLife. 2024 Apr 30;12:RP91927. doi: 10.7554/eLife.91927 (PMC11060718; doi:10.7554/eLife.91927)
Supplement: Figure 3—source data 3. [file elife-91927-fig3-data3.zip › Figure 3 source data3 part 1.zip!/Figure 3-Source data 3-part1/1071f2-1-CRISPResso_Report_ztwktvfp.zip!/CRISPResso_on_1071f2_4/2b.Nucleotide_percentage_quilt_around_sgRNA_gcagaaggatttgtaaatgg.pdf]

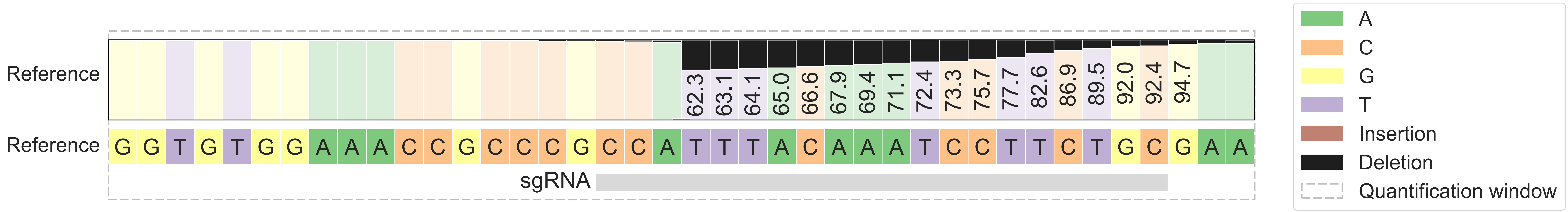

Reference
62.3
63.1
64.1
65.0
66.6
67.9
69.4
71.1
72.4
73.3
75.7
77.7
82.6
86.9
89.5
92.0
92.4
94.7
Reference
G G T G T G G A A A C C G C C C G C C A T T T A C A A A T C C T T C T G C G A A
sgRNA
A
C
G
T
Insertion
Deletion
Quantification window
